# Application of near-infrared spectroscopy for hay evaluation at different degrees of sample preparation

**DOI:** 10.5713/ab.23.0466

**Published:** 2024-02-28

**Authors:** Eun Chan Jeong, Kun Jun Han, Farhad Ahmadi, Yan Fen Li, Li Li Wang, Young Sang Yu, Jong Geun Kim

**Affiliations:** 1 Graduate School of International Agricultural Technology, Seoul National University, Pyeongchang 25354, Korea; 2 School of Plant, Environmental, and Soil Sciences, Louisiana State University, Agricultural Center, Baton Rouge, LA 70803, USA; 3 Research Institute of Eco-friendly Livestock Science, Institute of GreenBio Science Technology, Seoul National University, Pyeongchang 25354, Korea

**Keywords:** Hay Quality, Near-infrared Spectroscopy, Predictive Equation

## Abstract

**Objective:**

A study was conducted to quantify the performance differences of the near-infrared spectroscopy (NIRS) calibration models developed with different degrees of hay sample preparations.

**Methods:**

A total of 227 imported alfalfa (*Medicago sativa* L.) and another 360 imported timothy (*Phleum pratense* L.) hay samples were used to develop calibration models for nutrient value parameters such as moisture, neutral detergent fiber, acid detergent fiber, crude protein, and *in vitro* dry matter digestibility. Spectral data of hay samples prepared by milling into 1-mm particle size or unground were separately regressed against the wet chemistry results of the abovementioned parameters.

**Results:**

The performance of the developed NIRS calibration models was evaluated based on R^2^, standard error, and ratio percentage deviation (RPD). The models developed with ground hay were more robust and accurate than those with unground hay based on calibration model performance indexes such as R^2^ (coefficient of determination), standard error, and RPD. Although the R^2^ of calibration models was mainly greater than 0.90 across the feed value indexes, the R^2^ of cross-validations was much lower. The R^2^ of cross-validation varies depending on feed value indexes, which ranged from 0.61 to 0.81 in alfalfa, and from 0.62 to 0.95 in timothy. Estimation of feed values in imported hay can be achievable by the calibrated NIRS. However, the NIRS calibration models must be improved by including a broader range of imported hay samples in the modeling.

**Conclusion:**

Although the analysis accuracy of NIRS was substantially higher when calibration models were developed with ground samples, less sample preparation will be more advantageous for achieving rapid delivery of hay sample analysis results. Therefore, further research warrants investigating the level of sample preparations compromising analysis accuracy by NIRS.

## INTRODUCTION

Forage production in South Korea is limited primarily due to a lack of pasture and a rice-dominant agricultural production system. Therefore, the shortage of domestic forage supply is filled with imported hay [1]. Alfalfa and timothy are the most imported hay types, proportioning around 24.1% and 24.0% of the total imported hay, respectively [1]. Accurate nutrient concentrations in hay are critical in balancing ruminant animals' diets to ensure animal growth and productivity [2].

Unlike the wide range of quality and availability in domestically cultivated forage, imported hay is more standardized with moisture content and quality. However, even the hays baled in the same batch or field often present significant nutrient value variations. Since hay exporters collect hay from different sources, possible nutrient variation can occur through the distribution process of imported hay. The Korean National Institute of Animal Science forage research group tested hays imported between 2016 and 2018 for nutrient concentration and found substantial discrepancies between the labeled nutrient concentration and actual analysis results [3]. Therefore, quality assurance of hay seems to rely on a trust-based relationship between sellers and buyers. The quality of imported hay has not been critically monitored by South Korean agricultural administrations. Moreover, subjective quality estimations based on sensory or visual often cause complaints among livestock producers. Therefore, systemized forage quality evaluation measures are required to establish a reliable hay trade based on nutritional profile and price.

The forage quality evaluations are conventionally performed by wet-chemistry procedures, which are laborious and time-consuming from the sample preparation stage. Samples are supposed to be milled to 1-mm particle size using standard grinders because the size is critical for legume and grass particle passage through the rumen [4]; thus, the procedures are sometimes incompatible with routine commercial forage quality evaluations [5]. For example, *in vitro* digestibility analysis takes about a week to measure the potential degradability of forage biomass. However, livestock producers or forage sales traditionally accept total digestible nutrient or relative feed value as forage quality indexes, the calculated values based on analyzed fiber and protein concentration in forage. Therefore, marketing hay demands a reliable but quick analysis of forage samples. The near-infrared reflectance spectroscopy (NIRS) has been a quicker, less laborious, and more economical forage nutrient measurement option [5,6]. Since NIRS technology was adopted in the quality evaluation of agricultural products in the early 1960s, the development in hardware and mathematical algorithms made the NIRS-based analysis more reliable [7].

Although sample analysis is claimed to be chemically non-destructive and rapid, forage sample analysis by NIRS has been done after sample drying and milling. The standard forage sample drying condition has adopted temperatures between 55°C and 65°C for 72 hours. The rapid forage sample analysis still requires at least three days of sample preparation, which may limit immediate sample analysis capability at a commercial level. Moreover, there is a criticism that sample milling may alter the hay composition [6]. Bypassing the conventional sample preparation before NIRS scanning will save labor, time, and other expenses and possibly minimize sample preparation errors. Since immediate sample analysis improves daily nutrient management in cattle operations [6], some handheld NIR devices are commercialized for on-farm use. However, the accuracy and consistency of the devices are skeptical [8].

A study was designed to measure the differences in the calibration model developed with different sample preparations. Calibration models for NIRS were developed with the spectral data of unground hay or conventional ground sample scanning to compare the accuracy of imported hay analysis. The performance of the calibration models was also tested with external cross-validation hay samples prepared unground hay or ground hay samples.

## MATERIALS AND METHODS

### Hay sample collection and sample preparation for calibration model development

Hay samples with 227 alfalfa and 360 timothy were randomly collected from imported hay bales in different batches. Approximately 2 kg of each hay sample was divided into two portions to simulate sample preparations, such as ground (conventional forage sample preparation) or unground (minimum sample destruction) hay samples. Both portions of hay samples were dried at 55°C in a forced-air drying oven for more than 72 hours. The ground sample preparation was to mill hay samples into 2-mm particle size using a Wiley mill (Thomas Scientific, Swedesboro, NJ, USA) and then milled into 1-mm particle size using a cyclone mill. The unground hay was prepared by cutting hay samples roughly less than 10 cm long.

### Wet chemistry procedures

Hay samples milled to 1-mm particle size were used for wet chemistry analysis. Approximately 0.25 g of samples were used to determine total nitrogen concentration by Bremner [9] using an automated nitrogen analyzer (Euro Vector EA3000; EVISA Co., Ltd, Milan, Italy). The total N concentration was multiplied by 6.25 to estimate crude protein (CP) in samples. Neutral detergent fiber (NDF) and acid detergent fiber (ADF) were determined using an Ankom^2000^ fiber analyzer (Ankom Technology, Fairport, NY, USA) following the method of Van Soest et al [10]. The *in vitro* dry matter digestibility (IVDMD) was determined after the 48-h incubation of samples with a buffered-rumen fluid in an Ankom Daisy^II^ incubator (Ankom Technology, USA), according to Goering and Van Soest [11]. Rumen fluid was obtained from Holstein steers (*Bos taurus*).

### Spectral data collection using NIRS

Samples prepared by the conventional method (milling to 1-mm particle size) were packed evenly in a ring cup (diameter 85 mm) provided by the NIRS manufacturer (SpectraStar XT; Unity Scientific; KPM Analytics, Westborough, MA, USA) for NIRS scan. The unground hay samples were scanned using a large sample holder (diameter = 115 mm) provided by the same manufacturer. The spectrum was collected between 680 and 2,500 nm wavelength range with 2 nm interval. The absorbance was measured in reflectance (R) mode and then transformed into reciprocal natural logarithm reflectance (log 1/R), which is the first spectral pre-processing phase that results in better calibrations relative to reflectance [12]. Plots were created of log 1/R versus wavelength.

### Development of NIRS calibration model and model validation

The local calibration model of NIRS for the nutrient value parameters was developed using UCal software (version 4; Unity Scientific of KPM Analytics, USA). The sample population was structured based on *Mahalanobis* distance at 3.0 to detect outliers in spectral data [12]. For outlier detection in the laboratory dataset, the t-statistic value was set at 2.5. This statistic indicates that a value is an outlier if the difference between predicted and observed values exceeds 2.5 standard errors. The standard normal variate and detrend were used to correct the spectral scattering effect. Detrend correction has been reported to minimize the variations in the physical properties of NIRS spectra, including particle size and environment noise such as temperature [13]. Therefore, various mathematical treatments were also applied to correct the spectral imperfections. The mathematical treatment sets such as 1, 4, 4; 1, 8, 8; 1, 16, 16; 2, 4, 4; 2, 8, 8; 2, 16, 16; 3, 4, 4; 3, 8, 8; 3, 16, 16; 4, 4, 4; 4, 8, 8; and 4, 16, 16 were employed to the spectral data in the order of derivative order, derivative gap, and smoothing. The best spectral pretreatment was chosen for the lowest standard error and the highest coefficient of determination (R^2^) of cross-validation. The calibration models were generated using partial least squares regression, which has been identified as a regression method that retains most of the variation in the original values and prevents the overfitting of a calibration model [6]. The maximum number of factors was set at 16, and the cross-validation statistics included R^2^, standard error, and the ratio of prediction to deviation (RPD, calculated as the standard deviation of laboratory (observed) data divided by the standard error of prediction). The acceptable performance of the developed NIRS calibration models was determined based on R^2^ of cross-validation (>0.80) and SECrV close to the standard error of calibration (SEC). The RPD greater than 2 was considered acceptable as calibration models [14].

## RESULTS AND DISCUSSION

### Summary of nutrient constituents and correlation coefficients

Table 1 summarizes nutrient value parameters in alfalfa and timothy hays. The maximum moisture concentrations were less than 200 g/kg in hays, meaning all imported hay fell within the safe moisture range for storage without possible spoilage. The moisture ranges of alfalfa were slightly greater than timothy hay but not to a substantial degree. The alfalfa hay presented greater feed values of high CP concentration and IVDMD than timothy hay but low fiber concentrations, which agrees with Yu et al [15]. The standard deviations of the nutrient parameters were greater in alfalfa hay samples than in timothy, indicating the alfalfa quality had greater variation. Also, some hay's extremely low CP values indicate insufficient quality of the corresponding hay to support CP requirements for heifers or milking cows. The current CP, NDF, and ADF value ranges are similar to those reported for alfalfa samples collected in Spain [16]. However, the nutrient value in hay samples reflected significant differences from alfalfa samples collected from central, western, and northern New York (USA), as reported Berzaghi et al [17]. These differences could be reasoned from specific agronomic conditions such as growth stage, cutting height, genetics, and growing conditions [15]. For example, Rego et al [16] suggested differences in harvest number and growth stage as factors affecting leaf-to-stem ratio, resulting in significant variations in alfalfa CP content. Alfalfa grown under high temperatures produced lower digestibility biomass than grown under low temperatures due to greater accumulation potential of ADF and acid detergent lignin, while increasing temperatures resulted in a decreased N concentration in timothy but had an opposite effect in alfalfa [18]. Except for dry matter (DM), standard deviations were greater with the other nutrient parameters in alfalfa hay than timothy hay, indicating a possibility of more robust calibration model development from the alfalfa hay dataset.

### NIR spectra and calibration and cross-validation statistics

**Figure 1:**
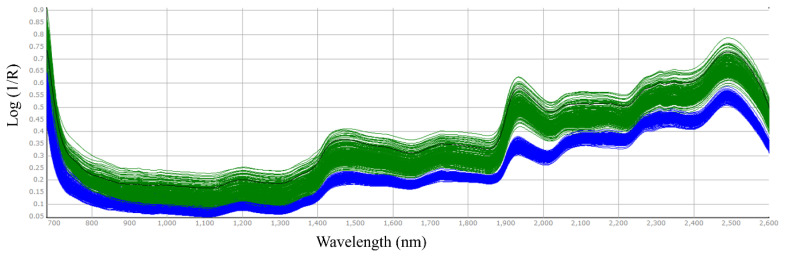
Spectra of alfalfa hay samples with uncut alfalfa hay (green colored) and 1-mm size milled alfalfa hay (blue color).

**Figure 2:**
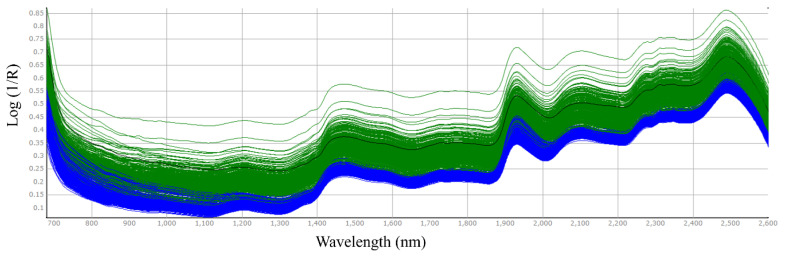
Spectra of timothy samples with unground hay (green colored) and 1-mm size milled hay (blue color).

The NIR spectra of alfalfa and timothy hays in whole or ground samples are shown in Figures 1 and 2, respectively. The hay spectra in unground hay exhibited more scattered spectrum patterns than those in ground samples. This pattern is consistent with the findings of Prananto et al [19], identifying more consistent NIRS spectral pattern of dried, ground leaf versus fresh, intact leaf. Peaks were detected at wavelengths around 1,200 nm, 1,400 to 1,500 nm, and 1,900 to 2,000 nm.

The calibration and cross-validation statistics of the nutritive value parameters are presented by unground and ground alfalfa (Table 2) and timothy hay (Table 3). The partial least squares regression of spectrum against wet chemistry data indicated higher parameter prediction accuracies of alfalfa and timothy hays when spectral data were obtained from ground sample preparation. This suggests that the NIRS detects the variations in nutrient parameters related to molecular bondings of alfalfa and timothy hays with less interruption than unground hay samples due to dense and uniform sample packing in the scanning device when samples were prepared with conventional 1 mm particle size. The more significant R^2^ drops between the comparison of calibration and cross-validation of unground hay samples indicated insufficient calibration with the reduced sample preparation and the necessity for including more samples and some standardized packing density at NIRS scanning [20].

Except for the moisture, the R^2^ of the nutrient parameters was equal to or greater than 0.9 in alfalfa hay. Timothy hay was above 0.9, except for IVDMD when the calibration models were developed with the spectral data acquired with ground samples. Among the nutrient parameters, moisture concentration was least predictable in alfalfa hay, while IVDMD was least in timothy. In all aspects, the nutrient parameter prediction models developed with unground hay indicated unreliable performance in prediction accuracy [6,14]. According to Stuth et al [21], the difference in forage particle size accounts for most spectral noise affecting calibration statistics. Particle size influences diffuse reflection, which is radiation reflection from a sample, and has been identified as a false principal factor in light scattering [20]. Barnes et al [22] also reported that differences in sample particle size altered surface scattering and radiation path length. Sample grinding resulted in more uniform distributions of plant materials, which probably exposed more consistent representations of chemistry in samples [12].

The current study demonstrated superior CP estimation performance in both hay species. Andueza et al [23] explained the high accuracy of CP by NIRS analysis with the increased diversity of the reference values. Another research also reported high accuracy of CP estimation (R^2^>0.95) due to the strong absorptions in N–H band in the NIR region [16]. As reported in [21,24], the calibration model development could be advantageous for a fitting when a wide range of CP concentrations is applied. There was also a report taking residual moisture in forages as another interfering factor with the quantification CP using NIRS. Moisture overshadowing may be prevalent at wavelengths where –N–H band vibration, a major component of proteins, may occur and potentially lower the precision of CP prediction in forages using NIRS [6]. In this experiment, the calibration statistics for CP were more accurate with ground timothy than ground alfalfa hay (R^2^ of cross-validations, 0.77 vs 0.95). Perhaps the R^2^ of moisture in the alfalfa calibration model may be related to the relatively lower accuracy of alfalfa CP calibration. This difference in CP calibration model performance between alfalfa and timothy hays agrees with earlier reports that confirmed the greater accuracy of the calibration statistics for grasses than legumes, primarily because of the variability in the leaf-to-stem ratio, which is also associated with variations in CP concentrations [25].

The non-uniform nature of fiber resulted from different chemical entities making up ADF and NDF in forage. Therefore, the absorption may occur across a wide range of spectral regions, potentially complicating the development of a partial least squares regression between laboratory and spectral data to define fiber fractions in forages [26]. A review of previous studies reporting forage quality prediction using NIRS indicated that fiber is one of the most estimated components after nitrogen [21,24]. Although NDF is not a simple chemical entity like nitrogen, variations in –O–H and –C–H bonds in NDF take DM of forage from 30% to 80%, which has been suggested as the main reason for the high performance of fiber prediction using NIRS even with its complicated entity [21]. In the current study, the R^2^ of the NDF was slightly lower than ADF due to the contribution of the hemicellulose component in NDF. However, the prediction models of the parameters were above 0.9, similar to a previous study with Italian ryegrass [27].

Digestibility is a more combined parameter affected mainly through cell wall components such as NDF, ADF, and lignin concentrations in samples. Therefore, even the prediction of digestibility in IVDMD is more complex than other nutrient parameters defined by chemical constituents [21]. However, like fiber and nitrogen, IVDMD prediction using NIRS has been achieved at a reasonable level in NIRS calibration modeling based on absorptions at 2,270 nm infrared region relevant to cellulose and lignin [24]. The prediction performance of IVDMD is usually more accurate in samples with a moisture level below 8% because of potential interference from moisture in forage samples with NIRS spectra [24]. Because organic compounds in the samples may contain O–H bond, which is also related to moisture, this bond potentially affects the spectra of hydrogen bonding in samples changing band position and width [28]. Therefore, the alterations of hydrogen bonding may result in substantial changes in the related NIR spectrum.

Both characteristics of nutrient value parameters and wet chemistry analysis potentially cause errors in IVDMD prediction of forage and feeds using NIRS [20]. Since NIRS calibration is based on accurate wet chemistry analysis results, consistency of the lab analysis procedure would be critical. For example, rumen inoculum can be a source of analysis errors in IVDMD caused by donor animals (species or genotype), daily feed, and rumen fluid collection time [29]. The complication of biochemical factors such as fiber and nitrogen digestibility and anti-quality factors may also contribute to variations in IVDMD analysis. The developed IVDMD prediction models exhibited low accuracy when compared to the accuracy (R^2^ cross-validation = 0.82) of Lundberg et al [30]. Since well-developed NIRS calibration models are possible only when accurate wet chemistry analysis results and properly collected spectral data are available, appropriate sample preparation and consistent sample scanning technique must be the critical components in forage sample analysis using NIRS [12].

As anticipated, the NIRS calibration model developed with ground alfalfa or timothy hay samples demonstrated greater R^2^ values when compared with unground hay samples (Tables 2 and 3). Since the study did not standardize the packing density of the unground hay samples in the sample scanning container of NIRS, there must be substantial variations in the spectral data of unground hay samples. The variations in the moisture concentration in samples can be another possible error source. Although the current study’s calibration models were developed with the spectral data of hay samples collected from multi-country origins at the calibration development, the NIRS calibration for imported hay may need to include broader quality hay samples. Therefore, consideration will be necessary to include more diverse hay samples produced across different origins, years, and production batches to upgrade the calibration models in the future continuously.

## CONCLUSION

Because of low accuracy, the NIRS calibration model for nutrient parameters cannot be developed with the spectral data obtained from scanning unground hay samples. However, rapid evaluation for imported hay quality is possible when NIRS calibration models are developed with the ground (1 mm particle size) hay samples. The calibrated NIRS could estimate CP most consistently and accurately among the tested nutrient parameters. The current setting could not estimate nutrient values of unground hay with NIRS. However, a more refined degree of sample preparation balancing the sample preparation efforts and accuracy of analysis results should be further studied with more refined settings.

## Figures and Tables

**Table 1:** Descriptive statistics of dry matter (g/kg) and nutrient concentration (g/kg DM) of imported alfalfa (*Medicago sativa*) and timothy (*Phleum pratense*) hays

Items	DM	CP	NDF	ADF	IVDMD
Alfalfa hay (n = 227)					
Minimum	828	85	351	230	526
Maximum	955	212	616	492	784
Mean	918	155	502	349	692
SD	12.4	26.3	37.1	35.8	42.1
Timothy hay (n = 360)					
Minimum	870	22	620	237	455
Maximum	950	115	725	434	703
Mean	922	53.8	674	383	673
SD	14.6	16.6	17.6	21.5	17.6

Each value is an average of three analytical replications.

Range = maximum – minimum.

DM, dry matter; CP, crude protein; NDF, neutral detergent fiber; ADF, acid detergent fiber; IVDMD, *in vitro* dry matter digestibility; SD, standard deviation.

**Table 2:** Calibration and cross-validation statistics of nutritive value parameters of imported alfalfa (*Medicago sativa*) hay prepared as a whole (<10 cm) or ground (1-mm particle size) samples.

Items	Nutrient parameter				

	DM	NDF	ADF	CP	IVDMD
Calibration	----------------------------------------------------------- Unground hay sample (10-cm stem) --------------------------------------------				
Mathematical treatment	3,16,16	3,4,4	2,4,4	1,16,16	1,4,4
Selected sample number	106	143	97	90	89
PLS terms^1)^	12	5	2	3	3
R^2^	0.74	0.89	0.68	0.42	0.52
SE	0.22	0.72	0.84	0.74	1.23
Cross-validation					
R^2^	0.42	0.43	0.32	0.19	0.18
SE	0.51	1.72	1.63	1.38	2.26
RPD^2)^	2.43	2.16	2.2	1.91	1.86
Calibration	--------------------------------------------------------- Ground hay (1-mm particle size) ------------------------------------------------------				
Mathematical treatment	2,16,16	1,8,8	4,16,16	4,16,16	4,16,16
Selected sample number	107	103	99	92	103
PLS terms^1)^	4	5	14	4	6
R^2^	0.84	0.92	0.97	0.9	0.95
SE	0.19	0.69	0.35	0.54	0.81
Cross-validation					
R^2^	0.61	0.77	0.81	0.77	0.79
SE	0.34	1.33	1.17	0.97	1.62
RPD^2)^	3.65	2.79	3.06	2.71	2.6

DM, dry matter; NDF, neutral detergent fiber; ADF, acid detergent fiber; CP, crude protein; IVDMD, *in vitro* dry matter digestibility; R^2^, coefficient of determination; SE, standard error.

1) PLS terms, number of factors in partial least square equation. The optimal number of terms was identified based on minimum SE and maximum R of calibration.

2) RPD, ratio percentage deviation, calculated as standard deviation divided by standard deviation of cross validation.

**Table 3:** Calibration and cross-validation statistics of nutritive value parameters of imported timothy (*Phleum pratense*) hay prepared as a whole (<10 cm) or ground (1-mm particle size) samples

Items	DM	NDF	ADF	CP	IVDMD
Calibration	--------------------------------------------------------------------- Unground hay -----------------------------------------------------------------------				
Mathematical treatment	3,8,8	1,8,8	1,16,16	3,8,8	1,16,16
Selected sample number	163	143	159	174	158
PLS terms^1)^	4	7	16	6	4
R^2^	0.48	0.38	0.75	0.95	0.61
SE	0.42	0.52	0.51	0.31	1.44
Cross-validation					
R^2^	0.18	0.14	0.42	0.83	0.32
SE	0.82	0.95	1.09	0.58	2.54
RPD^2)^	1.78	1.85	1.97	2.86	1.76
Calibration	------------------------------------------------------------------------- Ground hay -------------------------------------------------------------------------				
Mathematical treatment	3,16,16	1,4,4	4,16,16	1,16,16	1,8,8
Selected sample number	166	163	169	195	180
PLS terms^1)^	6	10	10	9	8
R^2^	0.95	0.94	0.94	0.98	0.84
SE	0.26	0.33	0.41	0.19	1.15
Cross-validation					
R^2^	0.88	0.75	0.77	0.95	0.62
SE	0.45	0.73	0.82	0.34	2.07
RPD^2)^	3.24	2.41	2.62	4.88	2.16

DM, dry matter; NDF, neutral detergent fiber; ADF, acid detergent fiber; CP, crude protein; IVDMD, *in vitro* dry matter digestibility; R^2^, coefficient of determination; SE, standard error.

1) PLS terms, number of factors in partial least square equation. The optimal number of terms was identified based on minimum SE and maximum R of calibration.

2) RPD, ratio percentage deviation, calculated as standard deviation divided by standard deviation of cross validation.
